# Transportation Insecurity, Chronic Illnesses, and Healthcare Access: Associations with Telehealth Utilization, Homecare Visits, and Well-Being

**DOI:** 10.3390/healthcare14142226

**Published:** 2026-07-22

**Authors:** Md Toushik Ahmed Niloy, Zeenat Kotval-K

**Affiliations:** School of Planning, Design & Construction, Michigan State University, 552 W. Circle Dr., East Lansing, MI 48840, USA; kotvalze@msu.edu

**Keywords:** transportation insecurity, chronic health conditions, healthcare accessibility, telehealth, homecare visits, well-being

## Abstract

**Background/Objectives:** Transportation insecurity and chronic health conditions can substantially influence healthcare access and alternative in-person healthcare utilization in urban areas. Alternative healthcare delivery models, such as telehealth services and professional homecare visits, may help address mobility-related barriers. This study examines how transportation insecurity, chronic health status, and healthcare access challenges are associated with homecare visits and telehealth utilization, while also assessing differences in health and well-being perceptions among individuals with and without chronic health conditions in Dallas, Texas, and Detroit, Michigan. **Methods:** A cross-sectional survey design was employed among 1649 respondents using a questionnaire. Binary logistic regression analyses were conducted to examine associations between transportation insecurity, chronic health status, healthcare access challenges, homecare visits, and telehealth utilization. Mann–Whitney U tests were used to compare perceived physical health and mental health between participants with and without chronic health conditions. **Results:** Transportation insecurity emerged as having a significant relationship with homecare visits, with transportation-insecure respondents being approximately 4.7 times more likely to receive homecare services than transportation-secure respondents. Transportation insecurity and healthcare access challenges were also significantly associated with greater telehealth utilization in Detroit and both cities combined. Participants without chronic health conditions reported significantly more favorable perceptions of physical and mental health than those with chronic conditions. Transportation affordability, lack of available rides from family or friends, and long waiting times were identified as the most common healthcare transportation barriers. **Conclusions:** Healthcare utilization and well-being outcomes are significantly associated with self-reported chronic health status and transportation insecurity. Expanding transportation assistance programs, strengthening homecare services, and improving telehealth availability may enhance healthcare access and support better health outcomes among transportation-disadvantaged and chronically ill populations.

## 1. Introduction

Individuals living with chronic health conditions generally require more frequent interaction with healthcare systems than healthier populations. Transportation insecurity refers to difficulties in obtaining reliable, affordable, safe, and convenient transportation for essential daily activities, including healthcare visits. As a result, transportation-insecure and chronically ill people are more likely to use healthcare delivery alternatives such as telehealth services and homecare visits from healthcare professionals [1]. This study is significant because it addresses the relationships between self-reported chronic health status, transportation insecurity, telehealth service utilization, homecare visits by healthcare professionals, and well-being outcomes. By integrating healthcare access, transportation, and community factors within a single analytical framework, this research contributes to the growing literature on social determinants of health and sustainable healthcare delivery.

### 1.1. Chronic Health Status, Telehealth, and Transportation Insecurity

Telehealth is particularly useful for patients with long-term illnesses who need regular follow-up but may face transportation mobility constraints or fatigue associated with disease burden [2]. Research has shown that remote healthcare delivery can improve access to care and patient satisfaction, particularly for populations requiring frequent clinical contact [3]. Transportation insecurity may significantly influence the relationship between chronic health status and telehealth utilization. Low-income individuals with chronic diseases often face transportation barriers. These obstacles can discourage attendance at in-person appointments and increase the number of missed visits or delayed treatment [4]. In such circumstances, telehealth/telemedicine may function as a substitute for face-to-face consultations by reducing the need for physical travel. A descriptive cross-sectional study was conducted using an online questionnaire, where logistic regression results showed that 63% of patients reported being satisfied with the telehealth services they received, while 37.1% expressed dissatisfaction [5]. Another study showed that those facing transportation challenges were 40% more likely to use telehealth compared to those without transportation challenges [6]. According to another study on patients’ perceptions of telemonitoring, telehealth appears to have been both an effective and efficient system. Almost 90% of patients reported feeling satisfied with their care and learned more about their current health condition using the telehealth service [7]. While prior research has generally reported positive associations between chronic health conditions, transportation barriers, and telehealth utilization, little is known about whether these relationships vary across communities with different socioeconomic and healthcare access characteristics. Consequently, important gaps remain in understanding how transportation insecurity, access to healthcare, and chronic health status interact to influence telehealth utilization patterns among vulnerable populations.

### 1.2. Chronic Health Status, Homecare Visits, and Transportation Insecurity

Homecare visits by professional healthcare providers have emerged as an important strategy to support chronically ill patients who face mobility limitations and transportation challenges. Home healthcare services include nursing care, physical therapy, occupational therapy, chronic disease monitoring, medication management, and other supportive services delivered directly within patients’ homes [8]. There are over 12,000 Medicare-certified home health agencies throughout the United States, with approximately 3 million Medicare Fee-for-Service (MFFS) beneficiaries using home healthcare [9]. Home care decreases costs, improves health outcomes, and reduces hospital stays. A case–control study at the Mount Sinai Health System found that Hospital at Home (n = 295) patients had lower hospital readmission rates than hospital inpatients recruited from emergency departments (n = 212) (8.6% vs. 15.6%) [10]. In another study, high-risk homebound older adults experienced difficulties accessing healthcare services due to transportation limitations, leading to increased reliance on homecare visits by healthcare professionals. The study found that post-discharge home visits and ongoing care coordination significantly reduced hospitalization rates from 1.38 (pre) to 0.74 (post), and lowered hospital readmission rates from 0.38 (pre) to 0.15 (post) [1]. Existing research has primarily focused on the clinical effectiveness of homecare services and their ability to reduce hospital utilization among chronically ill patients. In particular, important gaps remain regarding how transportation insecurity, chronic health status, and healthcare access challenges interact to influence access to homecare services.

### 1.3. Chronic Health Status and Adverse Physical and Mental Effects

Chronic illnesses impose long-term physical burdens and emotional stresses that deteriorate quality of life and worsen self-rated health outcomes over time [11]. A large cross-national study used World Health Survey data from 245,404 participants across 60 countries to examine the relationship between chronic disease and depression. Their analysis revealed that individuals with chronic diseases had significantly lower health scores than those without chronic conditions [12]. Using logistic regression analysis from the United States Health and Retirement Study (N = 18,483), a study found that a one standard deviation increase in chronic physical illness burden was associated with 1.34 times higher odds of major depression in later life [13]. Their findings indicated that individuals with long-term chronic illnesses were substantially more vulnerable to depressive symptoms and poor psychological outcomes than healthier populations. Despite substantial evidence linking chronic illness to poorer physical and mental health outcomes, limited research has examined its influence on overall perceptions of well-being. Disparities in access to healthcare among population groups should be taken into account when distributing limited healthcare resources to improve health equity for vulnerable populations, in accordance with international and national policy recommendations [14]. Evidence suggests that telehealth interventions can improve health outcomes by enhancing access to healthcare services, supporting disease management, increasing patient engagement, and improving psychological well-being, although their effectiveness may vary depending on intervention design, patient characteristics, healthcare settings, and contextual factors [15].

Although previous studies have examined transportation barriers, telehealth utilization, and healthcare accessibility independently, there is a lack of research investigating the effects of transportation insecurity on the utilization of telehealth and homecare services. Furthermore, few studies have examined whether these relationships differ according to chronic health status or across metropolitan areas with distinct transportation and healthcare systems. To address these gaps, this study integrates transportation insecurity, chronic health status, telehealth utilization, and homecare visits within a unified analytical framework, providing a more comprehensive understanding of healthcare accessibility across two metropolitan populations.

## 2. Materials and Methods

This study uses a quantitative, cross-sectional survey framework of analysis. To examine the transportation barriers affecting healthcare access among low-income and chronically ill populations, this research used questionnaire surveys. The final analytical sample consisted of 1649 respondents. Among them, 790 participants were recruited from Dallas, including 555 respondents with chronic conditions and 235 without chronic conditions. In Detroit, the sample included 859 respondents, of whom 626 had chronic conditions, and 233 did not have chronic conditions. This section begins with an introduction to the study areas. Subsequently, the paper presents the data collection procedures and survey instrument, followed by the analyses used in this study.

### 2.1. Study Areas

To investigate how transportation insecurity, chronic health conditions, and healthcare access challenges have an impact on telehealth utilization, healthcare professionals’ homecare visits, and overall health and well-being, this research considers the cities of Dallas, Texas, and Detroit, Michigan. According to healthcare community representatives in these areas, health disparities are further aggravated by a combination of factors, including concerns about personal safety, restricted access to personal vehicles, and inadequacies in public transportation services [16,17].

### 2.2. Survey Instrument and Data Collection

The data collection instruments consisted of a questionnaire designed and administered through the Qualtrics survey platform. This study was reviewed and determined to be exempt by the Michigan State University Institutional Review Board (MSU IRB; Study No. 00011399). This study used two panels to collect data responses in the Fall of 2025. A Qualtrics research panel was used to collect survey respondents from both cities (400 responses from each city). More information on the Qualtrics panel and recruitment is posted on their site [18]. A panel from Cloud Research was then used to gather responses from those people who had some form of chronic health condition (390 participants were recruited from Dallas, and 459 participants were recruited from Detroit). More information on the Cloud Research prime panel can be found on their website [19]. Using two panels from different companies helped mitigate a common respondent pool that might introduce bias. However, both survey questionnaires were very similar in content and used census characteristics for respondent quotas. Respondents were selected if they lived in either of the cities and if they had a chronic health condition (for the Cloud Research panel). To focus on specific population groups in this study, we asked for even sampling for gender (50% each), age distribution (one-third from 18–30 years, one-third from 30–50, and one-third over 50 years), and race (to try to mimic the non-white population in Detroit and the Hispanic population in Dallas) and most importantly, to aim for oversampling of the population earning less than $30,000 annually in household income from both cities; therefore, the respondent pool would not reflect the general population in both cities. The final data was not weighted to represent the city-specific general population. The survey instruments were organized into six primary sections:

(a) Demographic and Socioeconomic Characteristics: This section collected information on participants’ demographic and socioeconomic backgrounds, including age, gender, household income, educational attainment, employment status, race/ethnicity, and marital status.

(b) Transportation Security Index (TSI): Transportation insecurity was measured using the Transportation Security Index (TSI), a survey-based instrument developed by researchers at the University of Michigan to assess transportation security symptoms and their severity [20]. The TSI utilizes a three-point Likert response scale (Never, Sometimes, and Often) to measure the frequency of transportation-related challenges. Participants were asked to report on their transportation experiences during the previous 30 days [21].

(c) Transportation Access and Mobility Patterns: This section examined participants’ transportation accessibility and travel behaviors, including their primary mode of transportation, travel frequency, transportation expenses, challenges associated with public transit use, and the availability of accessible transportation options.

(d) Healthcare Access: Questions in this section assessed participants’ access to healthcare services, including the frequency of primary care visits, emergency room visits, urgent care utilization, receipt of professional homecare services, access to specialist care, appointment delays, and missed or rescheduled healthcare appointments resulting from transportation-related barriers.

(e) Transportation-Related Barriers and Challenges: Participants were asked to report their experiences with various transportation barriers affecting healthcare access. These included challenges related to personal and public transportation, such as travel distance to healthcare facilities, transportation costs, limited transit routes, language barriers, safety concerns, cleanliness issues, long waiting times, and accessibility limitations for individuals with physical disabilities or mobility impairments.

(f) Health Condition: This section evaluated participants’ overall health and well-being, including self-reported physical health, mental health, physical activity levels, and access to community resources. Participants also assessed their perceptions of community safety, neighborhood walkability, and bikeability using a five-point Likert scale ranging from strongly disagree to strongly agree. Additionally, respondents were asked to indicate whether they had any diagnosed chronic health conditions.

### 2.3. Treatment of Variables

#### 2.3.1. Transportation Security Index (TSI)

The abbreviated six-item Transportation Security Index (TSI) was used to assess the frequency of transportation-related challenges experienced by respondents. The items captured experiences such as missed or rescheduled appointments, inability to travel when needed, and unmet transportation needs. Collectively, these questions measured the functional, emotional, and social consequences of transportation insecurity, reflecting the extent to which mobility limitations influence healthcare access, routine activities, and social interactions. Each respondent was assigned a composite TSI score representing the severity of transportation insecurity experienced. The complete distribution of summed TSI scores, ranging from 0 to 12, was examined for analysis. To evaluate transportation insecurity, the following analytical procedures were conducted:

(a) Each possible TSI summed score was identified and tabulated.

(b) The frequency and percentage of respondents corresponding to each TSI score were calculated.

(c) The TSI scores were subsequently categorized into three levels of transportation insecurity [21]:

No Insecurity: Represents little to no reported transportation-related difficulties, corresponding to TSI scores ranging from 0 to 3.

Marginal Insecurity: Indicates a moderate degree of transportation challenges and constraints, corresponding to TSI scores ranging from 4 to 8.

High Insecurity: Reflects frequent and severe transportation barriers associated with substantial mobility disadvantages, corresponding to TSI scores ranging from 9 to 12.

For the logistic regression analysis, the marginal insecurity and high insecurity categories were combined into a single group to create a binary outcome variable, coded as 0 = No Insecurity and 1 = Marginal/High Insecurity.

#### 2.3.2. Transportation Access Challenges

This variable was created from a set of questions asked on the survey. The questions asked were “Have you faced any of the following challenges because of transportation barriers”? If the respondent answered “yes” to any of the following answer options, they were coded as a “Yes” for transportation access challenges. The sub-questions are (1) Missed an appointment with a primary care doctor; (2) Missed an appointment with a specialty doctor; (3) Missed any other health-related appointments; and (4) Missed picking up a prescription.

#### 2.3.3. Chronic Health Conditions

This variable was created from a single question on the survey: Do you have a chronic health condition (example: diabetes, high blood pressure, heart disease, lung disease)? Respondents who answered yes to this question were coded as having a chronic condition. Note that all references to chronic conditions in this paper refer to broadly defined, self-reported chronic conditions.

#### 2.3.4. Homecare Visits from Professionals

This variable was created from a single question on the survey: Have you received homecare visits from healthcare professionals due to issues with transportation? Respondents who answered yes to this question were coded as having used Homecare Visits (1 = used homecare visits, 0 = not used homecare visits).

#### 2.3.5. Utilized Telehealth Services

This variable was created from a single question on the survey: Have you used telehealth services for your healthcare appointments? Respondents who answered yes to this question were coded as having used telehealth services (1 = utilized telehealth, 0 = not utilized telehealth).

### 2.4. Research Questions and Hypotheses

This research study is guided by the following four research questions and three hypotheses to support the research objectives:

➤ Research Question 1: To what extent do transportation insecurity, self-reported chronic health status, and healthcare access challenges influence the receipt of homecare visits from healthcare professionals?

✓ Hypothesis 1: Individuals with higher levels of transportation insecurity, self-reported chronic health conditions, and healthcare access challenges are more likely to receive homecare visits from healthcare professionals than those without these risk factors.

➤ Research Question 2: How do transportation insecurity, self-reported chronic health status, and healthcare access challenges influence telehealth utilization across different cities?

✓ Hypothesis 2: Individuals with greater transportation insecurity and healthcare access challenges are significantly associated with increased telehealth utilization and exert a stronger effect on telehealth use than self-reported chronic health status across the two study cities.

➤ Research Question 3: Are there significant differences in well-being perceptions between individuals with and without chronic health conditions?

✓ Hypothesis 3: Individuals without chronic health conditions report significantly more favorable perceptions of physical health and mental health than individuals with chronic health conditions.

### 2.5. Analysis

To test the proposed hypotheses, this research applied binary logistic regressions and the Mann–Whitney U test. The research questions focus on the likelihood of utilizing telehealth services, receiving homecare visits by healthcare professionals, and perceptions of well-being. For Hypotheses 1 and 2, the dependent variables are receiving homecare visits and telehealth utilization, respectively. Here, the binary logistic regression method was selected because both dependent variables, homecare use and telehealth use, are dichotomous in nature (e.g., 1 = utilized service; 0 = did not utilize service). Moreover, binary logistic regression analyses were conducted to evaluate the effects of self-reported chronic health status (yes/no), Transportation Security Index (TSI) status (no insecurity vs. marginal/high insecurity), and healthcare access challenges (yes/no) on the likelihood of receiving homecare visits and utilizing telehealth services. Chronic health conditions, TSI binary (no insecurity vs. marginal/high insecurity), and healthcare access challenges are the independent variables for both Hypotheses 1 and 2. Distinct binary logistic regression models were applied to examine homecare and telehealth utilization for the pooled sample (both cities combined) as well as for Dallas and Detroit individually. The model estimates the log odds of the probability of the outcomes as a linear function of the predictor variables and is expressed as

(1) $$\ln\;(\frac{p}{1-p})=\beta_{0}+\sum_{i=1}^{k}\beta_{i}X_{i}$$

where *p* represents the probability of the outcome occurring, $\beta_{0}$ is the intercept, $\beta_{i}$ are the regression coefficients, and $X_{i}$ are the independent variables.

In this research study, several variables from the questionnaire survey were measured using ordinal response categories (e.g., Likert scale items assessing overall health well-being experiences, such as completely disagree, disagree, neutral, agree, and completely agree). The outcome variables were measured on ordinal Likert scales, and the comparison involved two independent groups (chronic and non-chronic health status) for the study areas. Because the outcome variables were measured on an ordinal scale and did not satisfy the assumptions required for parametric tests, a rank-based non-parametric approach was utilized to compare differences between groups. Therefore, hypothesis 3 was evaluated using the Mann–Whitney U test to compare mean rank scores of overall health well-being between individuals with and without chronic health conditions.

The analyses in this study differentiate between respondents with and without chronic health conditions to identify variations in access to healthcare challenges across health status groups. The analyses were conducted in IBM SPSS Statistics for Windows, version 29.0 (IBM Corp., Armonk, NY, USA) using a combination of inferential statistical techniques to determine whether significant relationships and differences exist among the key variables related to self-reported chronic health status, transportation security, and healthcare access challenges.

## 3. Results

The results of the statistical tests are presented in the following subsections, in which each hypothesis is examined for Dallas, Detroit, and the combined datasets (Dallas and Detroit together). Table 1 presents the demographic characteristics and selected healthcare utilization measures for participants from Dallas and Detroit. Dallas participants were more likely to be male, White, employed full-time, and married compared to participants in Detroit. In contrast, Detroit had a higher proportion of Black respondents, retirees, and individuals with health insurance coverage.

Detroit participants had a higher proportion of individuals with annual household incomes below $35,000 (55%) compared to Dallas (43%), suggesting greater financial constraints that may affect healthcare utilization decisions and access to alternative healthcare services. Regarding healthcare access and utilization, Dallas respondents were more likely to self-drive to primary care appointments and reported greater telehealth utilization (53% vs. 43%), whereas Detroit participants reported slightly higher rates of receiving homecare visits from healthcare professionals (21% vs. 19%). These demographic and socioeconomic differences may contribute to the variations in healthcare utilization patterns observed between the two study areas.

### 3.1. Homecare Visits from Healthcare Professionals (Hypothesis 1)

A logistic regression was first performed including the city variable, and the interaction between the city and each of the three independent variables to test for interaction effects. The city variable was coded as 1 for Detroit and 2 for Dallas. Detroit was used as the reference category. The results showed that the city and each of the three interaction terms were not significant, indicating that being from a particular city had no effect on the results of the logistic regression. Subsequently, the city and all the interaction terms were removed, and binary logistic regressions were carried out between homecare visits and the three independent variables: chronic conditions, access challenges, and transportation insecurity. The results from the initial analyses that included the city and its interaction terms are shown in the first part of Table 2. The second part of that table shows the regression results that include all socioeconomic variables. The fully adjusted regression model that included socioeconomic variables is presented to lay the context of how the three main variables of interest fare when all socioeconomic characteristics are taken into consideration. The results show that apart from TSI and access challenges, only ethnicity (Hispanic respondents had 2.6 times higher odds of receiving homecare visits than non-Hispanic respondents) and Education (respondents with less than a bachelor’s degree had lower odds of receiving homecare visits than those with a bachelor’s degree and higher) had significant associations with homecare visits.

The results from Table 3 show that in Dallas, the transportation insecurity status has a strong and significant relationship with homecare visits (Wald = 27.785, Exp(B) = 5.400), indicating that respondents experiencing transportation insecurity had 5.4 times higher odds of receiving homecare visits. Those with a chronic health condition had 40% lower odds of receiving homecare visits (Wald = 5.545, Exp(B) = 0.617), whereas access challenges do not show a statistically significant relationship with receiving homecare services. For respondents in Detroit, participants who have marginal or high insecurity had 4.225 times higher odds of receiving homecare visits compared to participants reporting no insecurity. In the case of access challenges, participants with access challenges had 1.450 times higher odds of receiving homecare visit services compared to participants without access challenges. Self-reported chronic health status had no significant association with receiving homecare visits.

For both cities’ datasets, transportation insecurity shows a strong and statistically significant association with homecare visits (Wald = 57.482, Exp(B) = 4.699), suggesting that individuals experiencing marginal or high transportation insecurity had 4.7 times higher odds of receiving homecare visits compared to those with no transportation insecurity. Access challenges are also significantly associated with homecare visits (Wald = 6.185, Exp(B) = 1.412), indicating that individuals experiencing healthcare access challenges had 1.4 times higher odds of receiving homecare services. In contrast, chronic health status shows a significant but inverse relationship (Wald = 4.829, Exp(B) = 0.731), suggesting that individuals with chronic conditions had about 27% lower odds of receiving homecare visits compared to those without chronic conditions.

Overall, logistic regression results indicate that transportation insecurity has the strongest and most consistent relationship with homecare visits. Respondents experiencing marginal or high transportation insecurity were significantly more likely to receive homecare services, suggesting that mobility barriers increase reliance on in-home care. Similarly, respondents with healthcare access challenges had higher odds of receiving homecare visits.

### 3.2. Using Telehealth Services (Hypothesis 2)

As carried out for hypothesis 1, a logistic regression using the city and all the interactions between the city and the three independent variables was performed. For telehealth usage, the city variable and its interaction with the TSI is significant, indicating that being from a city had its unique relationship with telehealth use, especially when it came to those who faced moderate to high transportation insecurity. Compared to Detroit, respondents from Dallas were significantly more likely to have used Telehealth services. This also showed up in the logistic regressions that were run without the city and the interaction terms. The results from the regression that included the city and its interaction terms are shown in Table 4. The second part of that table shows the regression results that include all socioeconomic variables. The fully adjusted regression model that included socioeconomic variables is presented to lay the context of how the three main variables of interest fare when all socioeconomic characteristics are taken into consideration. Results show that apart from TSI and access challenges, race (non-white respondents had 23% lower odds of utilizing telehealth services than white respondents), employment (those that work for pay have 1.335 times higher odds of utilizing telehealth than those who did not work for pay), and education (respondents with less than a bachelor’s degree had lower odds of utilizing telehealth services than those with a bachelor’s degree and higher) had significant associations with telehealth utilization.

In Dallas, self-reported chronic health status is significantly associated with telehealth utilization (Wald = 5.952, Exp(B) = 0.674), indicating that respondents with chronic conditions had 33% lower odds of using telehealth services compared to those without chronic conditions. Access challenges have a strong and significant relationship with telehealth (Wald = 16.092, Exp(B) = 1.965), suggesting that individuals who experience difficulties accessing healthcare services are nearly two times more likely to have used telehealth services. However, transportation insecurity does not show a statistically significant association with telehealth use in Dallas. In the case of Detroit, participants who have marginal or high insecurity had 1.741 higher odds of using telehealth services compared to participants reporting no insecurity. However, self-reported chronic health status and healthcare access challenges do not show statistically significant relationships with telehealth use in Detroit (see Table 5).

For both cities taken together, transportation insecurity shows a statistically significant relationship with telehealth utilization (Wald = 6.791, Exp(B) = 1.370), suggesting that individuals experiencing marginal or high transportation insecurity had 1.37 times (or 37%) higher odds of using telehealth services compared to those with no transportation insecurity. Additionally, healthcare access challenges show a strong and statistically significant relationship with telehealth use (Wald = 15.424, Exp(B) = 1.562), indicating that respondents who face difficulties accessing healthcare facilities had 1.56 times (or 56%) higher odds of using telehealth services. In contrast, self-reported chronic health status does not show a statistically significant relationship with telehealth utilization in the combined cities dataset.

In summary, logistic regression results show unique and varying results between the two study cities, individually and combined. Transportation insecurity and healthcare access challenges had significantly higher odds of telehealth utilization in the combined sample, indicating that telehealth may serve as an alternative when mobility or access barriers exist. In Dallas, telehealth use was significantly associated with healthcare access difficulties and self-reported chronic conditions. In contrast, Detroit results show that only transportation insecurity has a significant association with telehealth use.

### 3.3. Well-Being Perceptions (Hypothesis 3)

From a conceptual standpoint, chronic health conditions may influence individuals’ perceptions of their physical and mental health. The Mann–Whitney U test was employed in this study to examine differences in overall well-being conditions between participants with chronic health conditions and those without chronic health conditions in Dallas, Detroit, and both cities.

In Dallas, participants without chronic conditions reported higher mean ranks for physical health (476.23) compared to those with chronic conditions (361.32), and this difference was statistically significant (Z = −6.814 ***). Similarly, perceptions of mental health mean ranks were significantly different (Z = −5.556 ***) among participants without chronic conditions (462.50) compared to those with chronic conditions (367.13) (see Table 6). Respondents without chronic conditions exhibited significantly higher rank distributions for both physical health and mental health, indicating more favorable self-reported health outcomes among participants without chronic conditions.

Here, the consistently negative Z-values reflect that respondents with chronic conditions had lower rankings across all categories, while the significance levels confirm that these differences are unlikely to be due to chance. According to Cohen’s guidelines, the observed effect sizes suggest that the magnitude of these differences was small to moderate for physical health and small for mental health [22]. Overall, the Dallas results suggest that individuals without chronic health conditions consistently report better physical and mental health compared to individuals with chronic conditions.

Similar to the Dallas findings, the Detroit dataset demonstrated significant differences in the rank distributions of self-reported physical and mental health between respondents with and without chronic conditions. For physical and mental health, participants without chronic conditions reported substantially higher mean rank scores, 516.31 and 477.81, respectively, compared to those with chronic conditions, 397.87 and 412.21, respectively. Z-values for these two categories are statistically significant, indicating that individuals without chronic conditions reported better perceived physical and mental health (see Table 7). Consistent with Cohen’s guidelines [22], the effect size for physical health (0.221) was small to moderate, whereas the effect size for mental health (0.122) was small. Although these differences were statistically significant, their magnitudes suggest that chronic health status had a modest relationship with respondents’ perceived physical and mental health among the Detroit population.

The Detroit results indicate that chronic health status is primarily associated with individual health perceptions, such as physical and mental health. These findings suggest that the impact of chronic health conditions on well-being may vary across urban contexts. These differences may reflect variations in neighborhood environments, transportation accessibility, or healthcare infrastructure across the two cities.

Based on both cities’ datasets, in each category, participants without chronic health conditions reported higher mean rank scores than those with chronic conditions, suggesting more positive perceptions of well-being. For perceived physical health, participants without chronic conditions had a substantially higher mean rank (991.89) compared to participants with chronic conditions (758.87). The difference was statistically significant (Z = −9.372 ***). This means that non-chronic participants reported significantly better physical health perceptions than the chronic participants. Here, a large negative Z-value combined with a significant *p*-value means that the mean value of the chronic status group (group one) is significantly lower than the mean value of the group without chronic status (group two). In terms of negative directionality, group one in the comparison has a lower average value compared to group two (see Table 8).

A similar pattern was observed for mental health, where participants without chronic conditions reported higher mean rank scores (940.85) compared to those with chronic conditions (779.09). This difference was also statistically significant (Z = −6.430 ***). The effect size for physical health (0.231) represents a small-to-moderate effect, whereas the effect size for mental health (0.158) indicates a small effect, suggesting that chronic health status is associated with modest differences in perceived physical and mental health across the combined study population. It suggests that chronic health status is associated with differences in perceived mental well-being. In addition, it is observed that the Mann–Whitney U value for all categories is larger. The reason is that in categorical data (e.g., Likert scales 1–5), many participants often choose the same category, resulting in many tied ranks. High tie density can reduce the sensitivity of the test and lead to larger Mann–Whitney U values. Therefore, as sample sizes increase, the resulting Mann–Whitney U value statistic will naturally be larger, even if the groups are moderately different [23]. These findings suggest that in the combined dataset of both cities, individuals without chronic health conditions tend to report more favorable perceptions of their physical and mental health compared to those living with chronic conditions.

### 3.4. Specific Transportation Challenges in Access to Healthcare

This study also examines the specific transportation-related challenges faced by individuals who rely on public transit when accessing healthcare services. To better understand these challenges, respondents who reported difficulties in finding transportation to healthcare were asked to identify the primary reasons contributing to these difficulties. The following figure presents the distribution of reported challenges among public transit users, with comparisons between individuals with chronic health conditions and those without chronic conditions.

**Figure 1:**
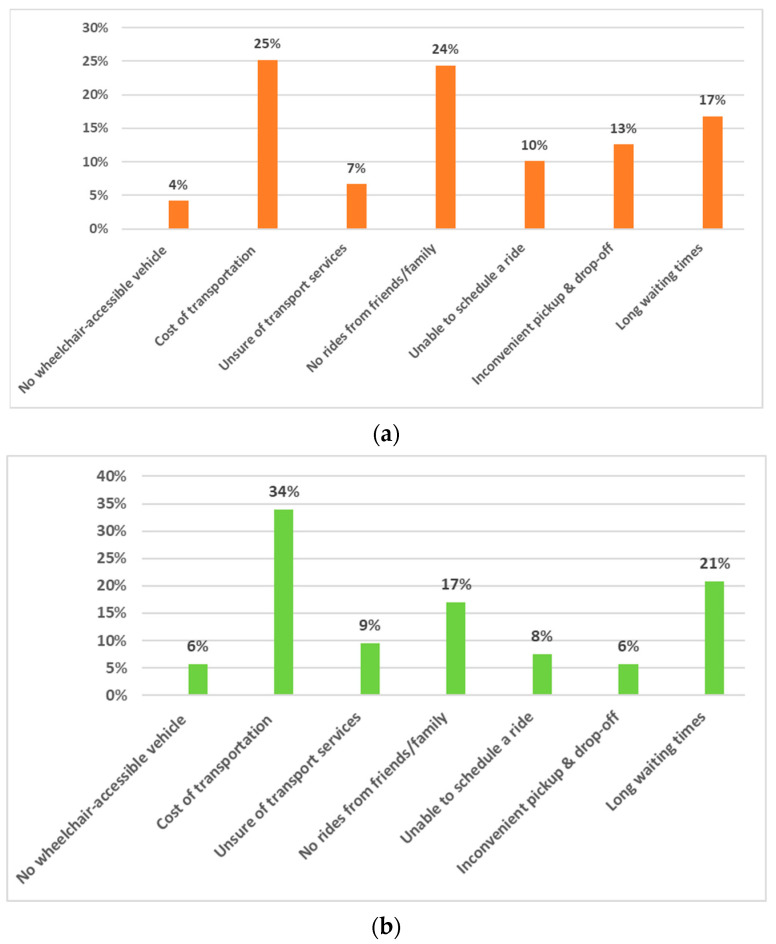
Reasons for difficulty finding transportation to healthcare in both cities. (**a**) All respondents with chronic condition; (**b**) all respondents with no chronic condition.

Figure 1 presents the reported reasons for difficulty in finding transportation to healthcare access among respondents in both cities, comparing individuals with and without chronic conditions. This figure illustrates that among respondents with chronic conditions, the most frequently reported challenge is the cost of transportation (25%), followed closely by no available rides from friends or family (24%). Other notable barriers include long waiting times (17%), inconvenient pickup and drop-off locations (13%), and difficulty scheduling a ride (10%). These results suggest that individuals with chronic health conditions often face financial and logistical difficulties when relying on transportation options to reach healthcare facilities.

For respondents without chronic conditions, the cost of transportation remains the most frequently cited barrier (34%), indicating that transportation affordability is a major concern for this group. This is followed by long waiting times (21%) and no available rides from friends or family (17%). Overall, transportation-related challenges, especially affordability, limited ride availability from family members, and service reliability, play a significant role in shaping healthcare accessibility among public transit users. These transportation challenges are significantly associated with individuals’ ability to reach healthcare facilities, thereby affecting timely healthcare access.

## 4. Discussion

The primary objective of this research was to examine how transportation insecurity, self-reported chronic health status, and healthcare accessibility are associated with homecare visits and telehealth services in two major U.S. cities: Dallas, Texas, and Detroit, Michigan. The inclusion of socioeconomic characteristics in the logistic regressions for homecare visits and telehealth pointed out that income was a consistent factor in the utilization of these services and that the main variables of interest (TSI, Access challenges, and Chronic health conditions) remained significant in the results, as they did when the regressions were carried out with only these three predictor variables. Results from the first research question demonstrated that across both cities, individuals categorized as transportation-insecure exhibit significantly higher odds of utilizing homecare services. This suggests that patients facing mobility constraints are substantially more likely to depend on healthcare professionals visiting them at home rather than accessing facility-based care. These findings strongly reinforce prior literature indicating that transportation-disadvantaged individuals are more likely to experience delayed care and to rely on non-traditional or emergency-based services [24]. A study in the US found that among older adults with chronic conditions, transportation dependency was a stronger predictor of homecare service needs than specific disease diagnoses. The study also found that 17% of participants who experienced transportation barriers on their appointment days utilized homecare visit services through the Medicare program [25]. People with chronic illnesses, lower-income households, transit-dependent populations, and those without access to private vehicles are disproportionately affected by mobility insecurity, which has a considerable impact on healthcare access outcomes among urban residents [26].

Transportation insecurity emerged as the most consistent predictor of receiving homecare visits, although its magnitude differed by city. Respondents experiencing transportation insecurity were 5.40 times more likely to receive homecare visits in Dallas, 4.23 times more likely in Detroit, and 4.70 times more likely in the combined sample than respondents without transportation insecurity, suggesting that transportation barriers substantially increase reliance on home-based healthcare services across both metropolitan areas. In contrast, chronic health status showed inconsistent associations between the city-specific analyses. While respondents with chronic conditions in Dallas were significantly less likely to receive homecare visits than those without chronic conditions, no statistically significant association was observed in Detroit, indicating that self-perceived chronic health status alone did not have a statistically consistent relationship with homecare utilization. For instance, a large population-based study found that individuals with chronic conditions accounted for a disproportionately high share of homecare service hours and visits, particularly those classified as long-term care patients or those requiring services over extended periods [27]. However, healthcare access challenges were significantly associated with homecare utilization in Detroit and the combined sample (those with access challenges were more likely to receive homecare visits than those without access challenges), whereas the association was not statistically significant in Dallas. Prior research supports this expectation. For example, a study found that an individual’s need alone is not sufficient to predict homecare service use, as structural and system-level factors significantly influence whether patients actually receive homecare services [28]. These findings suggest that transportation insecurity is more consistently associated with homecare utilization than chronic health status across both study regions, while the effects of healthcare access challenges appear to be related to local healthcare delivery systems and contextual differences between Dallas and Detroit.

The findings from the analyses for the second research question indicate that the use of telehealth services is more strongly associated with healthcare access challenges (in Dallas and the combined sample) and transportation insecurity (in Detroit and the combined sample) than with self-reported chronic health status alone (significant in Dallas only). Similarly, previous studies have shown that telehealth adoption increases when traditional healthcare access is constrained. For example, a national study found that individuals reporting barriers to in-person care, such as transportation issues, time constraints, and provider shortages, were significantly more likely to use telehealth services [29]. Transportation insecurity shows a more nuanced relationship with telehealth utilization. An earlier study highlighted that transportation is a major determinant of healthcare access, showing that those facing transportation challenges were 40% more likely to use telehealth than those without, even after controlling for socioeconomic and health-related factors [6]. Regarding chronic health status, in Dallas, participants with self-perceived chronic health conditions were less likely to utilize telehealth services than those without chronic conditions, a contrary finding to a large national study using Medicare data that found that beneficiaries with multiple chronic conditions had significantly higher rates of telehealth utilization than those without chronic illnesses [30]. Several factors may explain this discrepancy. Medicare beneficiaries are a population largely composed of older adults who often have established relationships with healthcare providers and may have greater access to structured chronic disease management programs that incorporate telehealth services. In contrast, the current study included a broader community-based sample from Dallas and Detroit, where telehealth access and utilization may vary considerably across socioeconomic groups, insurance coverage types, and levels of digital connectivity. Importantly, some chronic health conditions require regular in-person examinations, diagnostic testing, laboratory monitoring, or hands-on clinical assessments that cannot be fully replaced by virtual consultations. Consequently, individuals with chronic illnesses may continue to rely primarily on traditional face-to-face healthcare services despite the availability of telehealth options. Although this study focused on transportation insecurity, telehealth utilization may also be associated with other factors, including reliable internet access, digital literacy, availability of appropriate technology, and patients’ preferences and comfort with virtual healthcare services. Consequently, strategies aimed at improving telehealth utilization should address both transportation-related barriers and digital access challenges to ensure equitable healthcare access across diverse populations.

Individuals facing transportation barriers in Dallas and Detroit may be more effectively connected to healthcare systems through referral networks, care coordination programs, and community health initiatives that promote homecare and telehealth services. Transportation insecurity and healthcare inaccessibility may be a part of larger structural disadvantages in Dallas and Detroit, such as a lack of healthcare resources, digital inequality, and neighborhood disinvestment. Income disparities between the two study cities may partially explain the differences in telehealth utilization and homecare visits observed in the regression analyses. A larger proportion of Dallas respondents reported annual household incomes above $35,000 (56%) compared to respondents in Detroit (44%). Because telehealth services and professional homecare visits may involve direct out-of-pocket costs, particularly when insurance coverage is limited or unavailable, higher-income households may be better able to bear these expenses. As a result, Dallas respondents may have greater access to and utilization of alternative healthcare delivery options than Detroit respondents.

The findings from this study suggest that non-chronic participants reported significantly better physical and mental health perceptions than participants with self-reported chronic health conditions. The statistically significant differences in physical and mental health conditions observed in this study are also well supported by previous research. A study using the 2012 National Health Interview Survey (NHIS) dataset showed that adults with multiple chronic conditions experience greater functional impairment and lower physical well-being than individuals without chronic illness [31]. Importantly, adults with chronic conditions experience limitations in physical activities, including walking, climbing stairs, and performing routine daily tasks [32]. In addition, a large cross-national study involving over 245,000 participants from 60 countries found that individuals with chronic diseases were approximately two to three times more likely to experience depression compared to healthy individuals [12].

### 4.1. Policy Recommendations

Policymakers should expand transportation assistance programs that connect residents to healthcare services through subsidized transit passes, non-emergency medical transportation programs, and demand-responsive transit services. Such initiatives can reduce missed appointments, improve continuity of care, and lessen the burden placed on emergency healthcare services. Policymakers should also expand funding and reimbursement mechanisms that support homecare delivery, particularly for individuals with chronic illnesses, disabilities, and limited transportation options. Healthcare systems should develop proactive patient identification programs that use screening tools to identify transportation-insecure individuals who may benefit from homecare services. Such programs would enable healthcare providers to deliver preventive care, chronic disease management, medication monitoring, and post-discharge follow-up directly within patients’ homes [25].

In addition, healthcare organizations should implement digital literacy programs that assist patients in navigating telehealth platforms, scheduling virtual appointments, and accessing electronic health information [33]. Special attention should be given to older adults, low-income populations, and individuals with chronic health conditions who may encounter greater technological barriers. Integrating transportation insecurity screening into healthcare systems could improve healthcare system efficiency by reducing appointment cancellations and facilitating earlier intervention for chronic disease management.

### 4.2. Limitations of the Study

Despite providing important insights into homecare utilization, telehealth use, and well-being among individuals with and without chronic health conditions in Dallas and Detroit, this study has several limitations. First, the cross-sectional design limits the ability to establish causal relationships between transportation insecurity and healthcare utilization outcomes, as all variables were measured at a single point in time. Consequently, changes in transportation insecurity and healthcare utilization over time could not be evaluated. Second, the study relied on self-reported survey data, which may be affected by recall bias, social desirability bias, and reporting inaccuracies. As a result, the reported measures may not fully reflect participants’ actual experiences, potentially influencing the observed associations among the study variables. Future studies incorporating these factors and longitudinal designs would provide a more comprehensive understanding of healthcare utilization patterns. Chronic conditions are taken in the broad sense in this study, and this study does not delve into the clinical and functional limitations of each chronic condition. Furthermore, this study did not account for factors such as internet connectivity, digital literacy, technology availability, or patient preferences, all of which may be associated with telehealth utilization and should be incorporated into future investigations.

## 5. Conclusions

This study examined how transportation mobility barriers, self-reported chronic health status, and healthcare accessibility affect homecare visits, telehealth utilization, and health well-being among residents in Dallas, Texas, and Detroit, Michigan. Public transit dependence emerged as a major characteristic of the study population. Approximately 60% of respondents in both Dallas and Detroit reported using public transportation. Transportation cost, lack of rides from family/friends, and longer waiting times are the prime concerns represented as the most frequently reported reasons for avoiding public transit in both cities. These findings indicate that perceived difficulties in finding transportation to healthcare are related to mobility decisions among urban residents and may substantially reduce accessibility to healthcare for transit-dependent populations. Additionally, the higher proportion of vehicle ownership and self-driving behavior in Dallas may reduce transportation barriers for accessing healthcare facilities, while individuals experiencing transportation insecurity may rely more heavily on homecare and telehealth alternatives. Conversely, Detroit’s higher rates of lower-income households and slightly greater reliance on homecare services may indicate that transportation and socioeconomic barriers contribute to different patterns of healthcare utilization.

The findings suggest that telehealth utilization is closely associated with transportation-related barriers and healthcare accessibility challenges experienced by vulnerable populations. Respondents experiencing greater transportation insecurity demonstrated higher reliance on telehealth services compared to transportation-secure populations, indicating that virtual healthcare may function as an adaptive strategy for individuals facing mobility constraints. In addition, respondents in both cities who were experiencing greater transportation insecurity demonstrated an increased likelihood of utilizing homecare services compared to transportation-secure populations. However, the likelihood of utilizing homecare and telehealth services differed between the Dallas and Detroit respondents, suggesting that the relationship between transportation insecurity and healthcare utilization is associated with local contextual factors. These differences may reflect variations in healthcare infrastructure, transportation networks, socioeconomic conditions, service availability, and patterns of healthcare-seeking behavior across the two metropolitan areas. Policymakers should therefore consider regional characteristics when designing interventions aimed at improving healthcare access and reducing transportation insecurity. For chronically ill individuals requiring frequent medical monitoring, medication assistance, rehabilitation, or nursing care, home-based healthcare services may significantly improve healthcare continuity and reduce missed medical appointments.

Individuals living with chronic diseases appear to experience a multidimensional burden in which health limitations interact with environmental and transportation-related challenges to shape overall well-being. Findings strongly indicate that individuals with self-perceived chronic illnesses perceive their physical and mental health substantially less positively than those without chronic conditions. Additionally, transportation insecurity and healthcare access barriers may intensify physical and emotional distress among chronically ill populations by creating repeated uncertainty regarding access to treatment and healthcare continuity.

## Figures and Tables

**Table 1:** Demographic and selected behavioral characteristics of study participants.

Description	Percentage of Respondents	
	Dallas	Detroit
Male (Female)	48% (52%)	44% (56%)
White (Black)	67% (24%)	32% (64%)
Employed full-time (Retired)	54% (17%)	43% (19%)
Married (Never married)	44% (26%)	31% (41%)
One vehicle in household (0 vehicles in household)	44% (28%)	48% (29%)
Have health insurance (No health insurance)	84% (16%)	94% (6%)
Use Public Transit (Do not use public transit)	60% (40%)	59% (41%)
Respondents’ Annual Income under $35,000	43%	55%
Respondents’ Annual Income $35,000 to $49,999	12%	8%
Respondents’ Annual Income $50,000 to $74,999	19%	14%
Respondents’ Annual Income $75,000 to $99,999	11%	13%
Respondents’ Annual Income $100,000 and over	14%	9%
Drove themselves to Primary Care (Took public transit)	44% (24%)	39% (18%)
Received Homecare Visits	19%	21%
Utilized Telehealth Service	53%	43%

**Table 2:** Results from the binary logistic regression for likelihood of homecare visits with city interactions and then with socioeconomic variables included.

	B	S.E.	Wald	Sig.	Exp(B)
Regressions with City Interactions Nagelkerke R^2^ 0.112, N = 1649					
TSI_Binary	1.441	0.266	29.458	<0.001	4.225
Access Challenges	0.371	0.187	3.934	0.047	1.450
Chronic	−0.172	0.200	0.746	0.388	0.842
City	−0.167	0.404	0.170	0.680	0.846
City by TSI_Binary	0.245	0.416	0.348	0.555	1.278
Access Challenges by City	−0.055	0.279	0.038	0.845	0.947
Chronic by City	−0.310	0.286	1.173	0.279	0.734
Constant	−2.491	0.263	89.781	<0.001	0.083
**Fully adjusted model with socioeconomic variables included** **Nagelkerke R^2^ 0.234, N = 1649**					
	**B**	**S.E.**	**Wald**	**Sig.**	**Exp(B)**
TSI Binary (ref. no insecurity)	1.692	0.218	60.032	<0.001	5.432
Access Challenges (ref. no challenges)	−0.303	0.148	4.163	0.041	0.739
Chronic (Ref. no chronic)	−0.174	0.165	1.122	0.289	0.840
City (ref. Detroit)	−0.658	0.154	18.207	<0.001	0.518
Male (ref. females)	0.243	0.143	2.893	0.089	1.275
Under 70 yrs (ref. ≥ 70 yrs)	−0.517	0.309	2.794	0.095	0.597
Hispanic (ref. not Hispanic)	0.974	0.211	21.399	<0.001	2.649
Non-White (ref. White)	−0.187	0.157	1.414	0.234	0.830
Education (ref. bachelor’s and over)	−1.286	0.164	61.643	<0.001	0.276
Working for pay (ref. not working for pay)	−0.166	0.158	1.094	0.296	0.847
Any Income (ref. no income)	0.230	0.165	1.941	0.164	1.258
Vehicle (ref. 0 vehicles)	−0.294	0.168	3.058	0.080	0.745
No Ins. (ref. have insurance)	−0.080	0.226	0.125	0.724	0.923

**Table 3:** Summary table for the binary logistic regression results for homecare visits without city interactions.

Study Area	Independent Variables	Nagelkerke R Square	Regression Coefficients	Standard Errors	*p*- Values	Wald	95% CI	Exp (B)
Dallas N = 790	Chronic status	0.118	−0.482	0.205	0.019	5.545	0.413–0.922	0.617
	TSI Binary		1.686	0.320	<0.001	27.785	2.89–10.1	5.400
	Access Challenges		0.317	0.207	0.126	2.337	0.915–2.06	1.372
Detroit N = 859	Chronic status	0.103	−0.172	0.200	0.388	0.746	0.569–1.245	0.842
	TSI Binary		1.441	0.266	<0.001	29.458	2.511–7.109	4.225
	Access Challenges		0.371	0.187	0.047	3.934	1.004–2.092	1.450
Both Cities N = 1649	Chronic status	0.108	−0.313	0.142	0.028	4.829	0.553–0.967	0.731
	TSI Binary		1.547	0.204	<0.001	57.482	3.15–7.01	4.699
	Access Challenges		0.345	0.139	0.013	6.185	1.08–1.85	1.412

Note: Does not have a chronic condition, no insecurity, no access challenge; logistic regression testing for the likelihood of taking homecare visits from professionals; analytical N = 1649 for both cities, N = 790 for Dallas, N = 859 for Detroit.

**Table 4:** Results from the binary logistic regression for likelihood of utilizing telehealth with city interactions and then with socioeconomic variables included.

	B	S.E.	Wald	Sig.	Exp(B)
Regressions with City Interactions Nagelkerke R^2^ 0.054, N = 1649					
TSI_Binary	0.555	0.171	10.566	0.001	1.741
Access Challenges	0.260	0.157	2.758	0.097	1.297
Chronic	0.033	0.161	0.042	0.837	1.034
City	0.912	0.235	15.062	<0.001	2.489
City by TSI_Binary	−0.510	0.245	4.332	0.037	0.600
Access Challenges by City	0.415	0.230	3.264	0.071	1.515
Chronic by City	−0.427	0.228	3.508	0.061	0.652
Constant	−0.827	0.167	24.652	<0.001	0.437
**Fully adjusted model with socioeconomic variables included** **Nagelkerke R^2^ 0.114, N = 1649**					
	**B**	**S.E.**	**Wald**	**Sig.**	**Exp(B)**
TSI Binary (ref. No insecurity)	0.423	0.131	10.435	0.001	1.527
Access Challenges (ref. No challenges)	−0.405	0.119	11.584	<0.001	0.667
Chronic (Ref. No chronic)	0.057	0.124	0.215	0.643	1.059
City (ref. Detroit)	0.192	0.114	2.822	0.093	1.212
Male (ref. Females)	−0.063	0.108	0.340	0.560	0.939
Under 70 yrs (ref. ≥ 70 yrs)	−0.126	0.235	0.289	0.591	0.881
Hispanic (ref. Not Hispanic)	0.231	0.182	1.619	0.203	1.260
Non-White (ref. White)	−0.257	0.117	4.817	0.028	0.773
Education (ref. No schooling)	−0.697	0.127	30.135	<0.001	0.498
Working for pay (ref. not working for pay)	0.289	0.120	5.830	0.016	1.335
Any Income (ref. No income)	−0.208	0.123	2.832	0.092	0.813
Vehicle (ref. 0 vehicles)	−0.054	0.129	0.176	0.675	0.948
No Ins. (ref. have insurance)	−0.168	0.170	0.975	0.323	0.846

**Table 5:** Results from the binary logistic regression for using telehealth services without city interactions.

Study Area	Independent Variables	Nagelkerke R Square	Regression Coefficients	Standard Errors	*p*-Values	Wald	95% CI	Exp(B)
Dallas N = 790	Chronic status	0.043	−0.394	0.162	0.015	5.952	0.491–0.925	0.674
	TSI Binary		0.044	0.176	0.801	0.064	0.740–1.48	1.045
	Access Challenges		0.675	0.168	<0.001	16.092	1.41–2.73	1.965
Detroit N = 859	Chronic status	0.036	0.033	0.161	0.837	0.042	0.754–1.417	1.034
	TSI Binary		0.555	0.171	0.001	10.566	1.246–2.433	1.741
	Access Challenges		0.260	0.157	0.097	2.758	0.954–1.763	1.297
Both Cities N = 1649	Chronic status	0.032	−0.186	0.112	0.098	2.744	0.666−1.035	0.830
	TSI Binary		0.315	0.121	0.009	6.791	1.08–1.74	1.370
	Access Challenges		0.446	0.114	<0.001	15.424	1.25–1.95	1.562

Note: Does not have a chronic condition, no insecurity, no access challenge; [logistic regression testing for the likelihood of using telehealth services; analytical N = 1649 for both cities, N = 790 for Dallas, N = 859 for Detroit.

**Table 6:** Mann–Whitney U test results for the Dallas dataset.

Categories	Chronic Status	Mean Rank	Mann–Whitney U	Z-Value	Effect Size
Physical Health (N = 790)	Yes	361.32	46,242.0	−6.814 ***	0.242
	No	476.23			
Mental Health (N = 790)	Yes	367.13	49,468.5	−5.556 ***	0.197
	No	462.50			

(* *p* < 0.05; ** *p* < 0.01; *** *p* < 0.001) [analytical sample size in Dallas = 790].

**Table 7:** Mann–Whitney U test results for the Detroit dataset.

Categories	Chronic Status	Mean Rank	Mann–Whitney U	Z-Value	Effect Size
Physical Health (N = 859)	Yes	397.87	52,818.5	−6.480 ***	0.221
	No	516.31			
Mental Health (N = 859)	Yes	412.21	61,789.5	−3.562 ***	0.122
	No	477.81			

(* *p* < 0.05; ** *p* < 0.01; *** *p* < 0.001) [analytical sample size in Detroit = 859].

**Table 8:** Mann–Whitney U test results for both cities’ datasets.

Categories	Chronic Status	Mean Rank	Mann–Whitney U	Z-Value	Effect Size
Physical Health (N = 1649)	Yes	758.87	198,249.5	−9.372 ***	0.231
	No	991.89			
Mental Health (N = 1649)	Yes	779.09	222,137.0	−6.430 ***	0.158
	No	940.85			

(* *p* < 0.05; ** *p* < 0.01; *** *p* < 0.001) [analytical sample size in both cities = 1649].

## Data Availability

The datasets presented in this article are not readily available because the data are part of an ongoing study.

## Abbreviations

The following abbreviations are used in this manuscript:

NHIS	National Health Interview Survey
TSI	Transportation Security Index
MFFS	Medicare Fee-for-Service
